# Enhanced Preprocessing Approach Using Ensemble Machine Learning Algorithms for Detecting Liver Disease

**DOI:** 10.3390/biomedicines11020581

**Published:** 2023-02-16

**Authors:** Abdul Quadir Md, Sanika Kulkarni, Christy Jackson Joshua, Tejas Vaichole, Senthilkumar Mohan, Celestine Iwendi

**Affiliations:** 1 School of Computer Science and Engineering, Vellore Institute of Technology, Chennai 600127, India; 2 School of Information Technology and Engineering, Vellore Institute of Technology, Vellore 632014, India; 3 School of Creative Technologies, University of Bolton, Bolton BL3 5AB, UK

**Keywords:** liver disease, machine learning, multivariate imputation, feature scaling, ensemble learning, gradient boosting, XGBoost, bagging, random forest, extra tree classifier, stacking

## Abstract

There has been a sharp increase in liver disease globally, and many people are dying without even knowing that they have it. As a result of its limited symptoms, it is extremely difficult to detect liver disease until the very last stage. In the event of early detection, patients can begin treatment earlier, thereby saving their lives. It has become increasingly popular to use ensemble learning algorithms since they perform better than traditional machine learning algorithms. In this context, this paper proposes a novel architecture based on ensemble learning and enhanced preprocessing to predict liver disease using the Indian Liver Patient Dataset (ILPD). Six ensemble learning algorithms are applied to the ILPD, and their results are compared to those obtained with existing studies. The proposed model uses several data preprocessing methods, such as data balancing, feature scaling, and feature selection, to improve the accuracy with appropriate imputations. Multivariate imputation is applied to fill in missing values. On skewed columns, log1p transformation was applied, along with standardization, min–max scaling, maximum absolute scaling, and robust scaling techniques. The selection of features is carried out based on several methods including univariate selection, feature importance, and correlation matrix. These enhanced preprocessed data are trained on Gradient boosting, XGBoost, Bagging, Random Forest, Extra Tree, and Stacking ensemble learning algorithms. The results of the six models were compared with each other, as well as with the models used in other research works. The proposed model using extra tree classifier and random forest, outperformed the other methods with the highest testing accuracy of 91.82% and 86.06%, respectively, portraying our method as a real-world solution for detecting liver disease.

## 1. Introduction

A total of 264,193 deaths as a result of liver disease were reported in India in 2018, according to the latest World Health Organization data [1]. There are about 23.00 deaths per 100,000 people based on age-adjusted death rates for the population. With a weight of approximately 1.36 kg, the liver is the largest organ in the body. It has four lobes of differing sizes and shapes, and is dark reddish-brown in color. The liver is located right behind the diaphragm beneath the abdominal cavity. The hepatic artery and the portal vein are two major arteries that transport blood to the liver [2]. Its primary function is to eliminate poisonous and damaging compounds from the bloodstream before they are distributed to other regions of the body. WHO officials have identified liver disease as one of the most serious and deadly diseases [3]. Hepatitis infection, fatty liver, cirrhosis, liver fibrosis, high alcohol intake, drug exposure, and genetic anomalies can all cause liver disease [4]. A liver transplant is the only treatment option left if the liver has completely failed, and there is no way to recover it. Timely identification of liver illnesses can aid in therapy and speedy recovery. The phases of liver disease are: healthy, fibrosis, cirrhosis, and the last stage is cancer. Detecting liver disease in its early stages can be difficult, even after there is significant damage to liver tissue. This would lead to failure to provide proper treatment and drugs. An early diagnosis of the disease is crucial to preventing this and saving the patient’s life. Internal bleeding, dry mouth, constipation, and stomach pain are a few signs of liver disease that can affect the digestive system [3]. Some other signs include brain and nervous system anomalies such as loss of memory, numbness, and fainting, as well as skin concerns such as yellow skin, spider veins, and feet redness. Visiting a doctor regularly, getting vaccinated, drinking less soda and alcohol, exercising regularly, and keeping your weight in check can prevent liver diseases. The advancement of artificial intelligence has led to the development of numerous machine learning algorithms which enhance the accuracy and effectiveness of diagnosing and prognosticating liver disease [4].

In many automatic medical diagnostic tools, classification approaches are particularly common. Due to the fact that liver diseases do not manifest until the organ is partially damaged, it is difficult to detect early [5]. The presence of enzymes in the blood can be used to identify liver disease [5]. Furthermore, mobile devices are increasingly being utilized to track the health of humans. In this case, it is also necessary to use automatic classification algorithms. Mobile and online technologies capable of automatically identifying liver illnesses can be used to reduce patient wait times with liver specialists such as endocrinologists.

The remainder of the paper is organized as follows: In Section 2, the literature survey of liver disease classification and detection is presented. The problem statement is thoroughly explained in Section 3. A detailed explanation of the proposed architecture, algorithms, and preprocessing is provided in Section 4. The experimental setup and evaluation results has been given in Section 5. Section 6 discusses the conclusion and future work. 

## 2. Literature Survey

In this section, various machine learning methods applied to classify liver diseases are discussed. Machine learning models such as the support vector machine (SVM), logistic regression, naive Bayes, decision tree (DT), random forest, k-nearest neighbor (KNN), artificial neural network (ANN), etc., are used for liver disease classification. Bendi et al. applied various machine learning models to two datasets. They were the Andhra Pradesh (AP) liver dataset (Indian liver dataset) and the UCLA liver dataset [6]. The machine learning models used were naive Bayes, decision tree (C4.5), backward propagation, k-nearest neighbor, and support vector machine. It was found that k-nearest neighbor, backward propagation, and support vector machine provided better results compared with other models. It was also concluded that the AP liver dataset is better than UCLA for all models. In another study, Bendi et al. introduced a modified rotation forest algorithm to accurately classify liver diseases. Analyzing the combination of classification algorithms and feature selection techniques [7]. With the modified rotation forest algorithm applied to the UCI liver dataset, the multilayer perceptron (MLP) classification algorithm was used, and with the ILPD dataset that had the nearest neighbors with generalized distance functions and correlation-based feature selection, the random subset feature selection technique was used. It was observed that the multi-layer perceptron algorithm on the UCI liver dataset provided better results than neural net on the Indian liver dataset.

Yugal et al. proposed a rule-based model to classify data into various types of liver disorders using machine learning approaches [8]. The model used machine learning algorithms such as SVM, rule induction (RI), decision tree, naive Bayes, and ANN using the k-fold cross-validation methodology. Among all the models, the decision tree with a rule-based classification algorithm had better accuracy. They also created their dataset with 12 attributes and 583 records. Heba et al. used a decision tree model to predict an individual’s liver fibrosis degree [9]. It showed that using decision trees results in good classification accuracy. Liver disease disorders were analyzed using classification techniques such as C4.5, naive Bayes, SVM, neural network, and logistic regression [10]. The C4.5 model on the AP dataset proved to be better compared with the other models and the UCLA dataset. In a study conducted by Somaya et al., clinical biomarkers and mathematical models were used to predict advanced fibrosis in chronic hepatitis C patients [11]. For training the model, the data were divided into two sets according to the METAVIR score. They are (1) mild to moderate fibrosis (F0–F2), and (2) advanced fibrosis (F3–F4). The development of decision trees, genetic algorithms, particle swarm optimization, and multilinear regression models for advanced fibrosis prediction was performed. It was discovered that advanced fibrosis was statistically associated with age, platelet count, AST, and albumin.

Sumedh et al. classified liver diseases using SVM and backpropagation models [12]. The models were trained on the UCI repository dataset. Between both models, backpropagation performed better. Han Ma et al. evaluated an optimal predictive model to detect non-alcoholic fatty liver disease (NAFLD) [13]. The model was developed using data from individuals gathered during a health assessment at Zhejiang University’s First Affiliated Hospital. Among the 11 different models, the Bayesian network model performed the best. An interactive graphical user interface was created to help the medical community diagnose liver disease in patients [14]. Based on 583 patients from the Indian Liver Patient Dataset, the model was trained on 10 different attributes. In this study, various classification algorithms such as Logistic regression, KNN, SVM, and ANN are compared to see which delivers the best results. In comparison to other models, ANN was shown to have higher accuracy. Sivakumar et al. used another algorithm called C4.5 decision tree on the UCI repository using its 15 life quality attributes [15]. This work compared the performance of C4.5 with the k-means clustering algorithm. C4.5 recorded better precision values.

Vasan et al. also implemented using the UCI repository dataset [16]. The first step of this research work involved the application of a min–max algorithm to the original liver patient dataset. PSO feature selection is used in the second phase to demarcate relevant qualities. The entire normalized datasets of liver patients can then be used to extract the subset of critical liver-related data. After this stage, categorization algorithms are used in the third phase for comparisons and categorizations. It was found that the J48 algorithm performs best when it comes to feature selection. Vyshali et al. applied classification methods such as decision tree, linear discriminant analysis, SVM fine gaussian, and logistic regression [17]. The dataset consisted of laboratory data from 584 patients. The dataset contains 10 features that help in detecting liver diseases. The classification result on logistic regression was better than other models. Sateesh et al. worked on the ILPD for liver disease classification [18]. The random forest model was used for classification with various preprocessing techniques. The preprocessing technique was used for balancing the unbalanced data. Model refinement was carried out by hyperparameter tuning using grid search and feature selection. The work mostly focused on classification using random forest; thus, it cannot determine whether the selected model is best. Geetha et al. aimed to augment the perceived nature of liver disease using machine learning techniques [19]. The work mainly focused on algorithms that can classify healthy people from the liver dataset. The dataset used was the Indian Liver dataset. SVM and logistic regression were used for prediction. SVM gave better accuracy compared with logistic regression. 

Rong-Ho Lin employed machine learning models such as classification and regression tree (CART) and case-based reasoning (CBR) for liver disease classification [20]. To treat a new liver disease, doctors can use rules taken from CART for identifying liver disorders, whereas CBR can obtain the most comparable case from the case base for solving the problem. A study of risk factors concerning liver disease and predicting liver diseases was conducted using screening data accumulated from 1994 to 2001 [21]. It was shown that the risk factors useful for detecting liver disease are also useful for detecting liver cancer. It made use of logistic regression, decision tree, and ANN for predicting liver disorders. In comparison to a model employing current screening test data estimators, a neural network with a growth curve estimator outperformed the latter. It was also concluded that the sensitivity value improved by using the growth curve estimator. The findings of the [22] study showed that classification models predicted fatty liver disease in patients using minimum clinical parameters. Among other models tested, random forest models gave better accuracy. The strategy used led to more insights into real-world clinical practice and helped physicians to identify FLD (fatty liver disease) successfully. The drawback of this work includes using only one dataset.

## 3. Problem Statement

The liver is a very vital organ of the human body. Its failure can be fatal, and the only solution is a transplant within a given time. Various features such as total bilirubin, gender, age, SGPT, ALP, Albumin, etc., can be used for the early detection of liver illnesses in a person. Several research works discussed above make use of these features to detect liver disease. Though many machine learning classification-based algorithms are used in the literature, they have some drawbacks. In most existing works, only simple machine learning models are used, and ensemble models are not used. There are various data pre-processing methods that can be useful for improving results. These methods have not been explored as well. Additionally, many research works failed to employ efficient feature selection and transformation methods. To tackle this issue, the research makes use of various ensemble machine learning algorithms such as boosting, stacking, bagging, etc., to obtain better results. Furthermore, enhanced data pre-processing methods are applied with appropriate feature scaling and selection procedures to increase the model’s performance.

## 4. Proposed Architecture Using Ensemble Learning with Enhanced Preprocessing

An overview of the datasets, the proposed work, architecture, and algorithms used for liver disease classification are presented in this section.

### 4.1. Dataset to Perform Liver Disease Classification

**Figure 1:**
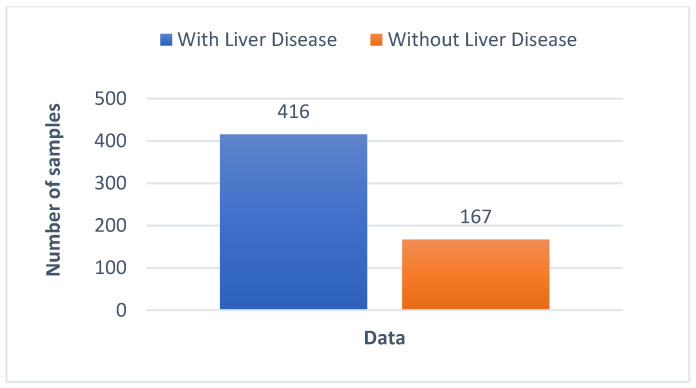
Dataset distribution graph.

The UCI machine learning repository’s Indian Liver Patient Dataset (ILPD) is used to perform liver disease classification. [23]. It contains 11 columns that have 10 features and a target variable. The features are age, gender, total bilirubin (TB), direct bilirubin (DB), total proteins (TP), albumin (ALB), albumin and globulin ratio (A/G), alamine aminotransferase (SGPT), aspartate aminotransferase (SGOT), and alkaline phosphotase (Alkphos). The characteristics of all the features for the patients are tabulated in Table 1. The output variable has two classes denoting patients with liver disease and those without. The dataset contains 583 records of patients collected from Andhra Pradesh’s North East region in India. The distribution of patients with and without liver disease is shown in Figure 1. 

### 4.2. Methodology and Architecture to Classify Liver and Non-Liver Diseases

**Figure 2:**
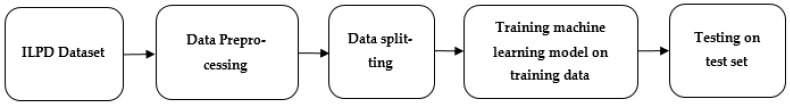
The proposed architecture.

In this subsection, the methodology used to classify liver and non-liver diseases is discussed. The dataset is first preprocessed using various techniques. The dataset is then split into training and test sets in order to train and assess the machine learning models. Machine learning models are trained on the created training set in order to classify the liver disease. The trained model is then tested on the test set and its performance is assessed using various metrics. The architecture of the above method is illustrated in Figure 2.

#### 4.2.1. Data Preprocessing

**Figure 3:**
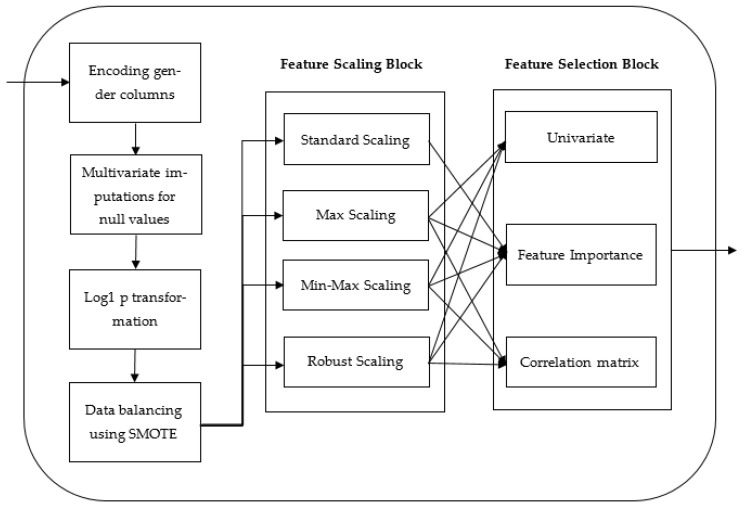
The proposed preprocessing block to classify liver disease.

The data were preprocessed using methods such as data encoding, data imputation, transforming skewed data, data balancing, feature scaling, and feature selection techniques, respectively. The above techniques are discussed in detail below. The overall architecture of the preprocessing steps is shown in Figure 3.

##### Data Encoding

It is necessary to convert categorical data into numerical values before training various models. This conversion is carried out using data encoding. In the Indian Liver dataset, there is only one categorical feature, which is gender. Gender columns contain female and male classes which are encoded to 0 and 1, respectively.

##### Data Imputation

Sometimes, the dataset contains missing values or null values. This is handled by either dropping the records with missing values or by using various imputation techniques. Imputations are of two types: univariate and multivariate. In univariate feature imputation approaches, the missing values of a particular feature are imputed using only the non-missing values of that feature [24]. Whereas in multivariate imputation, the missing values are estimated using all the features in the dataset. In the proposed work, the multivariate feature imputation is used using the IterativeImputer class of the sklearn library. It uses a regressor to predict the missing values. It is one of the best imputation techniques. 

##### Transforming Skewed Data

**Figure 4:**
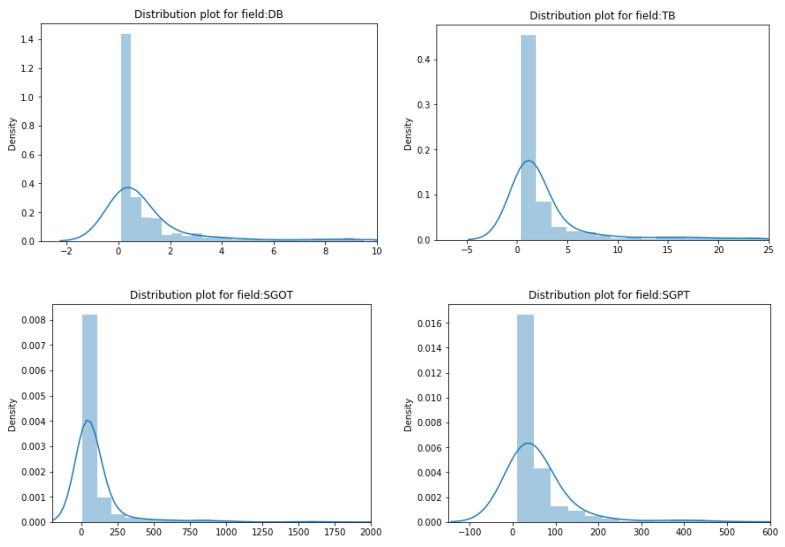

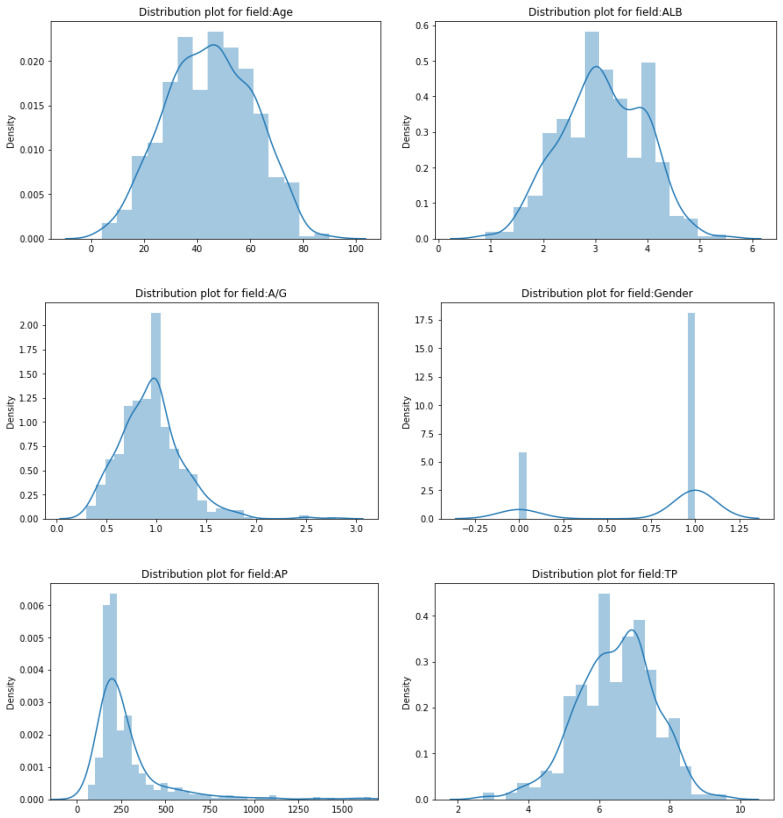
Observing the skewness of columns.

Distribution curves can be plotted to check whether the data is skewed or not. When the distribution curve of the data is distorted towards either the left or right side, then it is said to be skewed. Skewed data can affect performance by violating model assumptions or by affecting the interpretation of feature importances [25]. In the ILPD dataset, the features that are skewed are: ‘A/G’, ‘TB’, ‘AP’, ‘SGPT’, ‘DB’, and ‘SGOT’. There are various transformations available for handling the skewed data, but in this work, the ‘log1p’ transformation is used. It effectively helps to balance the distribution of the curve. The formula used for the ‘log1p’ transformation is given below Equation (1). The skewness of columns can be observed in Figure 4.

(1) $$X_{new}=log\left(1+X\right)$$

##### Data Balancing

This technique is used for when each output class’s number of data records available is comparably different. There are 167 records without liver disease and 416 records with the condition in the ILPD dataset. Data balance is essential since there is a large discrepancy in the number of records accessible for each class. To balance the dataset, the minority class, i.e., the class without liver disease was up-sampled to 416 records. After resampling, the total number of records in the dataset was 832. To balance the data, a resample function from the sklearn library is used. 

##### Feature Scaling

Feature scaling is a method for standardizing the independent features present in the data in a specific range [26,27]. It is one of the important steps for handling highly varying values. If the dataset is not scaled, regardless of the units, the larger values tend to be weighted higher and the smaller values lower by the machine learning models. Due to this reason, within the proposed work, different feature scaling methods are tried. They are as follows:

- Min–max normalization: This feature scaling method involves shifting and rescaling values to make them fall between 0 and 1. This technique is prone to outliers. The formula used is given in Equation (2).

(2) $$X_{new}=\frac{X-X_{min}}{X_{max}-X_{min}}$$

- Maximum absolute scaling: After applying this technique to features, its value ranges between −1 and +1. In this method, the values in a feature are divided by the absolute max value, as shown in Equation (3).

(3) $$X_{new}=\frac{X}{X_{maxabs}}$$

- Standardization: In standardization, the z value is calculated so the values are rescaled to have a distribution with 0 mean value and variance equal to 1 [26]. The formula used for the standardization is given in Equation (4).

(4) $$X_{new}=\frac{X-X_{mean}}{\sigma }$$

- Robust scaling: It is a feature scaling technique that is robust to outliers. In this method, the feature values are subtracted from their median and divided by the Inter-Quartile Range (IQR) value of that feature. IQR is the difference between Q1 (first quartile) and Q3 (third quartile). The robust scaling formula is given in Equation (5).

(5) $$X_{new}=\frac{X-X_{median}}{IQR}$$

##### Feature Selection

The feature selection method involves choosing a subset of all the available features that are more pertinent and contribute significantly to the target variable. The input features are reduced to improve the performance of the model, and sometimes to reduce computational costs. The strength of the association between the feature and the target variable is assessed using a variety of statistical approaches for feature selection. Some of the methods which are often used and was also used in this work are discussed below.

- Univariate feature selection: Univariate statistical tests are used in this strategy to determine the important features. In this, the relationship of a single feature is analyzed with the target variable ignoring other features. Hence, it is called univariate feature selection. From all the scores, features with top scores are selected. There are three tests used for feature selection in this work using the sklearn library. They are the chi-squared test, F-test, and mutual_info_classif test. The chi-squared test is used only for non-negative features and classes. It gauges the interdependence of stochastic variables [28]. The F-test, which is also known as the one-way ANOVA test, is based on the ANOVA F-value. The mutual information is computed for the discrete target variable in the mutual_info_classif test. Mutual information (MI), which evaluates the interdependence between two random variables, is a non-negative value [29].
- Feature importance: The feature importances of each feature of the dataset can be obtained for the target variable using the models. Each data feature is given a score; the higher the score, the more meaningful the feature. To obtain the feature importances of the models, it is trained on the dataset first. Based on the training, the scores are decided. Usually, tree-based classification models are used. In this work, models such as extra tree classifier, random forest, and LGBM classifier were used. All of these models are ensemble models.
- Correlation coefficient matrix: Correlation is used to determine the relationship between the features or the output variable. It measures the linear relationship between variables. The correlation coefficient can be positive (the output variable value increases as one feature value increases), negative (the output variable value decreases as one feature value increases), or zero (no relation between variables) [30]. The correlation matrix is a matrix containing the correlation value of each feature with every other feature in the dataset including the target. Ideally, features selected should be highly correlated to the target variable and not related to each other, otherwise the feature will not add any additional information. Hence, if two features are correlated, we can remove one of them. Typically, the correlation between characteristics is determined using Pearson’s correlation coefficient.

#### 4.2.2. Machine Learning Algorithms to Predict Liver Disease Using Enhanced Preprocessing

This research work evaluates the performance of ensemble-based machine learning algorithms on the ILPD (Indian Liver Patient Dataset) and compares their results. The Ensemble technique is a unique approach in which we combine multiple machine learning models of the same or different types such as decision tree, logistic regression, support vector machines, etc., to carry out prediction [31]. The models used in ensemble models are called base estimators or base learners. There are many reasons to use ensemble models over traditional models. A few reasons are mentioned below.

- Performance: A single model may not be able to give reliable results. Combining multiple models helps to increase prediction accuracy [32].
- Robustness: An ensemble helps in reducing the spread in the average performance of the machine learning model [32].
- Low variance: Ensembles help in reducing the variance (error) of the prediction by combining multiple models [32].

One model might not be able to forecast a dataset’s outcomes to the best of its ability. Therefore, simple machine learning models have limitations, and it is difficult to create a model with great accuracy [31]. If multiple models are combined, then the accuracy is boosted. Ensembles work on the mechanism of aggregation of output from individual models in such a way that model error is reduced, and generalization is maintained [31]. The algorithms employed in this research work have been thoroughly discussed in detail in the following sections. 

##### Gradient Boosting Classification Algorithm to Predict Liver Disease

In order to create a powerful regression or classification model, the gradient boosting classifier combines a number of weak learning models [33]. Decision trees are frequently used in gradient boosting. Due to their proficiency in classifying challenging datasets, gradient boosting models are becoming more and more popular, and have recently prevailed in a number of Kaggle data science competitions [33].

Gradient boosting classification has three main components as shown in Algorithm 1.

- Loss function: It determines how well a model is doing a prediction. More loss means the model could do better and vice versa [34]. Gradient descent is used to minimize this loss function value.
- Weak learner: A weak learner classifies data very poorly and can be comparable to random guessing. It has a high rate of errors. Usually, decision trees are used in this [34].
- Additive model: In this approach, trees are added iteratively and sequentially one at a time. After each iteration, the model is usually closer to the actual target [34].

**Algorithm 1** Gradient Boosting to Predict Liver Disease**Input:**     Training set record **Output:**   Class of record (liver disease or no liver disease) ***Generating Algorithm Begin***     **Step 1:** Calculate the initial log(odds) for the entire dataset       $log\left(odds\right)=\frac{+ve\;Class}{-ve\;Class}$      **Step 2:** Calculate the initially predicted probability for each record       $P=\frac{e^{log\left(odds\;\right)}}{1+e^{log\left(odds\right)\;}}$        *If the value is greater than 0.5*
**then**
*positive* class *else negative class.*     **Step 3:** Calculate the Residual for each record       R=Observed−Predicted      **Step 4:** Build a decision tree with leaves as residuals     **Step 5:** Calculate the output value of the leaf for each record        *O/P value =*
$\frac{\sum R}{\sum \;P\;X\;\left(1-P\right)}$      **Step 6:** Calculate the updated log(odds)       *log(odds) = log(odds) + (* γ X *o/p value)*
     **Step 7:** Calculate the updated predicted probability for each record       *Repeat steps 3 to 8 till residuals are small or till the number of trees specified*     **Step 8: Calculate the** testing **probability of each record**       *Step 8.1: Calculate log(odds)*           *log(odds) = log(odds) + ∑* γ *× o/p value of leaf*       *Step 8.2: Calculate the predicted probability* ***End***

##### XGBoosting Classification Algorithm to Predict Liver Disease

Similar to the gradient boosting algorithm, XGBoost uses gradient descent to enhance weak learners. However, XGBoost improves due to system optimization and algorithmic upgrades [35]. The system optimization applied in XGBoost are Parallelization, Tree Pruning and Hardware. The algorithmic enhancements applied in XGBoost are weighted quantile sketch, Regularization, Cross-validation, and Sparsity awareness.

##### Bagging Classification Algorithm to Predict Liver Disease

Bagging (bootstrap aggregation) is a classification technique that reduces the variance of prediction by taking the average of multiple predictions together [36]. Subsets called bootstrap samples (samples with replacement) are created from the main dataset, and the different base estimators are trained on these subsets [36]. This is called row sampling with replacement. The voting method (majority) is used in the case of classification for aggregating the prediction of different classifiers [36]. The variance decreases and the model’s performance rises by averaging the results [36]. Base classifiers such as decision tree SVM, etc. can be used. Algorithm 2 depicts the bagging approach to predict liver disease.
**Algorithm 2** Bagging to Predict Liver Disease**Input:**     Training set record **Output:**   Class of record (liver disease or no liver disease) ***Generating Algorithm Begin***     **Step 1:** Split data into bootstrap subsets equal to the number of classifiers say n taking all features     **Step 2:** Train n subsets on n base estimators, respectively     **Step 3:** Testing       *Step 3.1: Calculate the output of the test record on each base learner*       *Step 3.2: Calculate the final predicted value by using the voting method* ***End***

##### Random Forest Classification Algorithm to Predict Liver Disease 

Random forest classification algorithm is a type of Bagging method in which all the base learners are decision trees, and data samples are split by replacement. Random feature sampling is also applied. The best split approach is used while splitting the data. Decision tree is a weak learner and using multiple decision trees together has helped gain better results. Algorithm 3 depicts the random forest classification approach to predict liver disease.
**Algorithm 3** Random Forest Classification to Predict Liver Disease**Input:**     Training set record **Output:**   Class of record (liver disease or no liver disease) ***Generating Algorithm Begin***     **Step 1:** Split data into subsets equal to the number of classifiers say n with random feature selection and best split     **Step 2:** Train n subsets on n decision trees, respectively     **Step 3:** Testing       *Step 3.1: Calculate the output of the test record on each base learner*       *Step 3.2: Calculate the final predicted value by using the voting method* ***End***

##### Extra Tree Classification Algorithm to Predict Liver Disease 

Extra tree classification algorithm is an extended version of random forest with some variations. Similar to random forest, all the base learners are decision trees, but data samples are split randomly without replacement. Hence, instead of using the best split random split approach is used. Features are split randomly similar to random forest. This algorithm has given better results on noisy datasets compared with the random forest approach. Algorithm 4 depicts the extra tree classification algorithm to predict liver disease.
**Algorithm 4** Extra Tree Classification to Predict Liver Disease**Input:**     Training set record **Output:**   Class of record (liver disease or no liver disease) ***Generating Algorithm Begin***     **Step 1:** Randomly split data into subsets equal to the number of classifiers say n with random feature selection and random-split     **Step 2:** Train *n* subsets on *n* decision trees, respectively     **Step 3:** Testing         *Step 3.1: Calculate the output of the test record on each base learner*         *Step 3.2: Calculate the final predicted value by using the voting method* ***End***

##### Ensemble Stacking Classification Algorithm to Predict Liver Disease 

Stacking algorithms base estimators use the entire training dataset during training [36]. Once these base learners are trained, a meta-learner is assembled from the different models, and the base learner’s output is used for the training of the meta-learners [36]. A heterogeneous ensemble is created by this approach as the base learners are usually different algorithms [36]. This work uses ensembles models as the base model for stacking as shown in Algorithm 5.
**Algorithm 5** Ensemble Stacking Classification to Predict Liver Disease**Input:**     Training set record **Output:**   Class of record (liver disease or no liver disease) ***Generating Algorithm Begin***     **Step 1:** Train the entire dataset on n-base learners     **Step 2:** Feed output of base learners to meta learner        *Base learners used: extra tree classifier, random forest, and xgboost*     **Step 3:** Train meta learner on-base learner output        *Meta learner used: logistic regression*     **Step 4:** Testing            *Step 4.1: Pass each record through base learners*            *Step 4.2: Feed output of base learners to meta learner*            *Step 4.3: Meta-learner output gives final prediction* ***End***

In the above subsections, various feature scaling, feature selection methods, and machine learning algorithms are discussed. First, the data are split into training and testing so that every model receives the same train test split. Then, for each of the six algorithms, a default model is trained, and the best pair of feature scaling and feature selection combination is found. This is carried out by training and checking the model’s training accuracy on all combinations of feature scaling and selection pairs. In order to obtain the best feature scaling and selection pair, the optimal hyper-parameters are obtained using grid search with 10-fold cross validation. GridSearchCV from the sklearn library was used for this purpose. It uses all the specified hyper-parameters in various combinations and then calculates the performance for each. The best value for the hyper-parameters is then chosen. We performed training on hyper-parameters obtained from grid search, as well as default hyper-parameters. The best out of the two was chosen for comparison. The hyperparameter optimization carried out for all the models is given in Table 2. Finally, the models are trained by passing this list of optimal parameters to each model. Parameters such as the number of estimators, learning rate, etc., are passed to the models. The best result for each model evaluated on the test set (external validation) is then stored. This proposed method is named as enhanced preprocessing.

## 5. Evaluation and Analysis

In this section, details about the experiments performed on the Indian Liver Patient Dataset to classify liver disease are discussed. The metrics used for evaluation and the results obtained are explained. In the last sub-section, the results are compared with the existing works.

### 5.1. Experimental Setup

The experiments performed in the proposed work were carried out on the local system with Windows 10 operating system. The local system had the following specifications: 8 Gb RAM, intel i5–9th generation processor, and NVidia GTX1650 graphics card. No external GPUs were used. All the code was written in python language in jupyter notebook. Visual Studio code was used for running the notebooks. Various popular machine learning libraries such as pandas, numpy, sklearn, seaborn, etc., are used for the execution of the work.

### 5.2. Evaluation Metrics

Evaluation metrics help in determining how well-trained models perform on unseen test data. All ensemble models were tested using precision, accuracy, recall, specificity, and F1-scores on the test dataset. The following metrics have been described in the previous papers [37,38] as shown in Equations (6)–(10). Apart from these, the AUC (area under the curve) and ROC (Receiver operating characteristics) are also calculated with the help of graphs. The ROC is a probability curve, whereas the AUC is a measure of separability. As AUC increases, the model becomes more accurate at differentiating classes. The following terms help in calculating these metrics which are given in Equations (6)–(10).

- True Positive (*TP*)—when positive values are predicted as positive.
- True Negative (*TN*)—when negative values are predicted as negative.
- False Positive (*FP*)—when negative values are predicted as positive.
- False Negative (*FN*)—when positive values are predicted as negative.

(6) $$Accuracy=\frac{TP+TN}{TP+TN+FP+FN}$$

(7) $$Precision\;\left(P\right)=\frac{TP}{TP+FP}$$

(8) $$Recall\;\left(R\right)=\frac{TP}{TP+FN}$$

(9) $$F1-score=\frac{2\times \left(P\times R\right)}{\left(P+R\right)}$$

(10) $$Specificity=\frac{TN}{TN+FP}$$

### 5.3. Experimental Results

**Figure 5:**
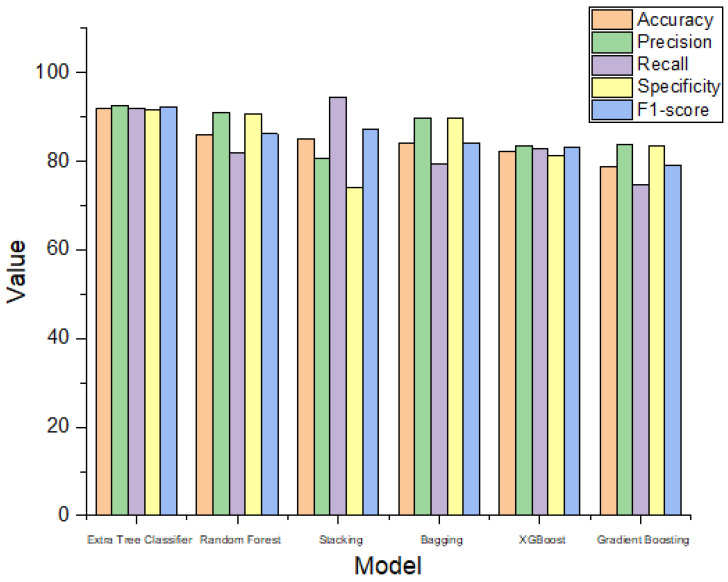
Metrics comparison between different models.

**Figure 6:**
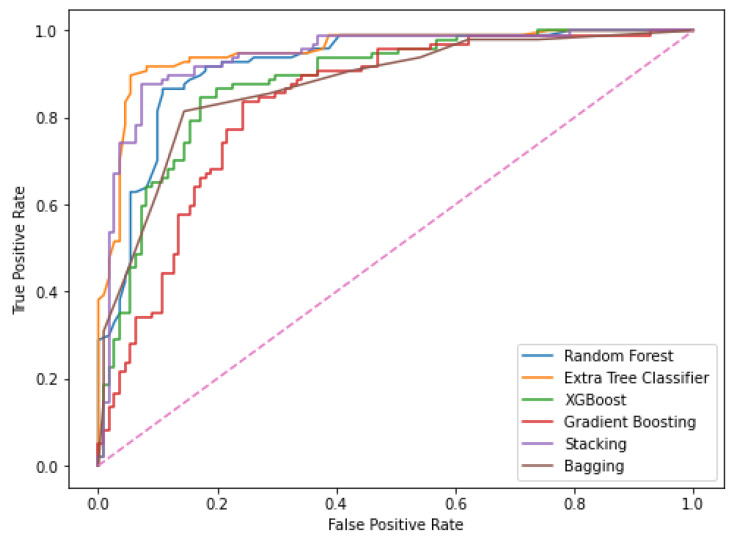
ROC curve of models.

**Figure 7:**
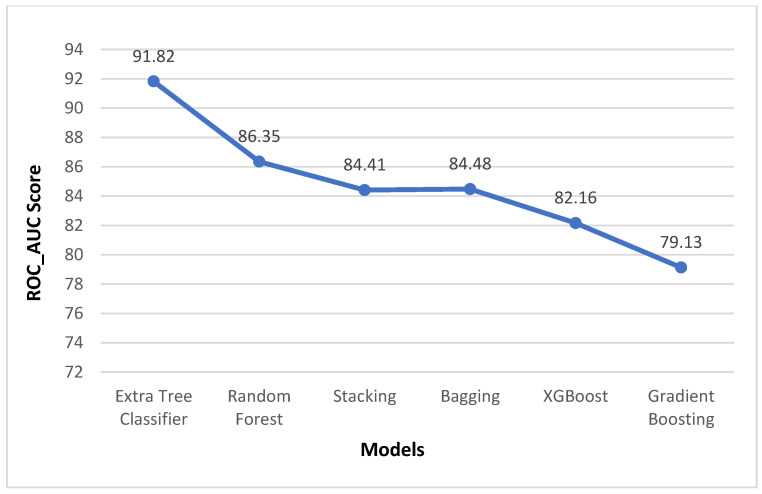
Metrics comparison graph of ROC_AUC.

**Figure 8:**
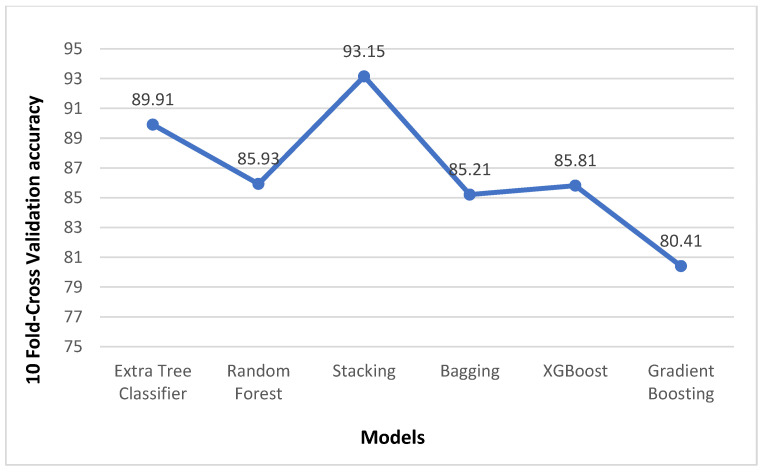
Comparison of 10-fold cross validation accuracy of different Models.

The results obtained for the liver disease classification on the ILPD dataset using various machine learning models have been illustrated above in Table 3 and Table 4. The uncertainty in the model has been represented in terms of confidence interval (CI) using bootstrapping in Table 3. The comparison graphs of these models for different metrics can be seen in Figure 5. The results shown above in Table 3 and Table 4 are obtained after applying enhanced preprocessing techniques on all the models. The receiver operating characteristic (ROC) curves have been plotted in Figure 6. According to that combination of feature selection, feature scaling preprocessing techniques with imputation and data balancing were used. The preprocessed data were finally trained and tested on all the six models. The results obtained uses the evaluation metrics such as accuracy, precision, recall, specificity, F1-score, ROC–AUC, and 10-fold cross validation. The graphs for ROC–AUC and 10-fold cross validation are given in Figure 7 and Figure 8, respectively. Among them, extra tree classifier had the highest testing accuracy of 91.82% followed by random forest with an accuracy of 86.06%. Gradient boosting had the lowest accuracy. When the models were tested with 10-fold, the cross validation stacking model had the highest accuracy of 93.15% and lowest accuracy of 80.41% for the gradient boosting model. When all the metrics are taken into consideration, the extra tree classifier shows the best performance, whereas the gradient boosting shows the worst. 

#### 5.3.1. Statistical Test Results

**Figure 9:**
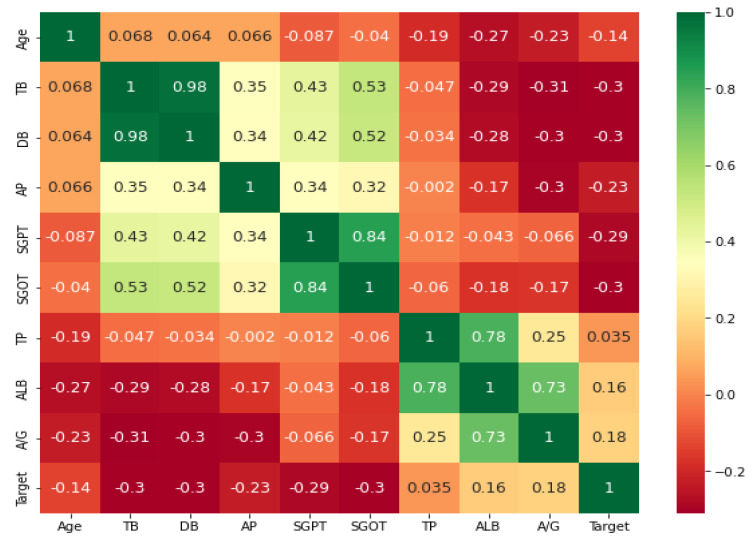
Correlation matrix.

Statistical tests such as Pearson’s Correlation test, chi-squared contingency test and analysis of variance (ANOVA) F-test have been performed on the data. The correlation matrix has been shown in Figure 9. It can be inferred from the test that features DB and TB, SGOT and SGPT, ALB and TP, ALB and A/G are highly correlated. The chi-squared test was performed between the gender and the target variable as both are categorical types of data. As the p-value of the test obtained is 6.55%, the null hypothesis is not rejected at 95% level of confidence. As per the null hypothesis, liver disease and gender are independent. The ANOVA F-test scores are shown in Table 5. As per the ANOVA F-test, the score obtained between Target variable and features such as DB, TB, SGOT, SGPT is very high, whereas the score obtained between the target variable and feature such as gender, the TP, is low. The higher the score, the more the features are dependent on the target variable. 

**Figure 10:**
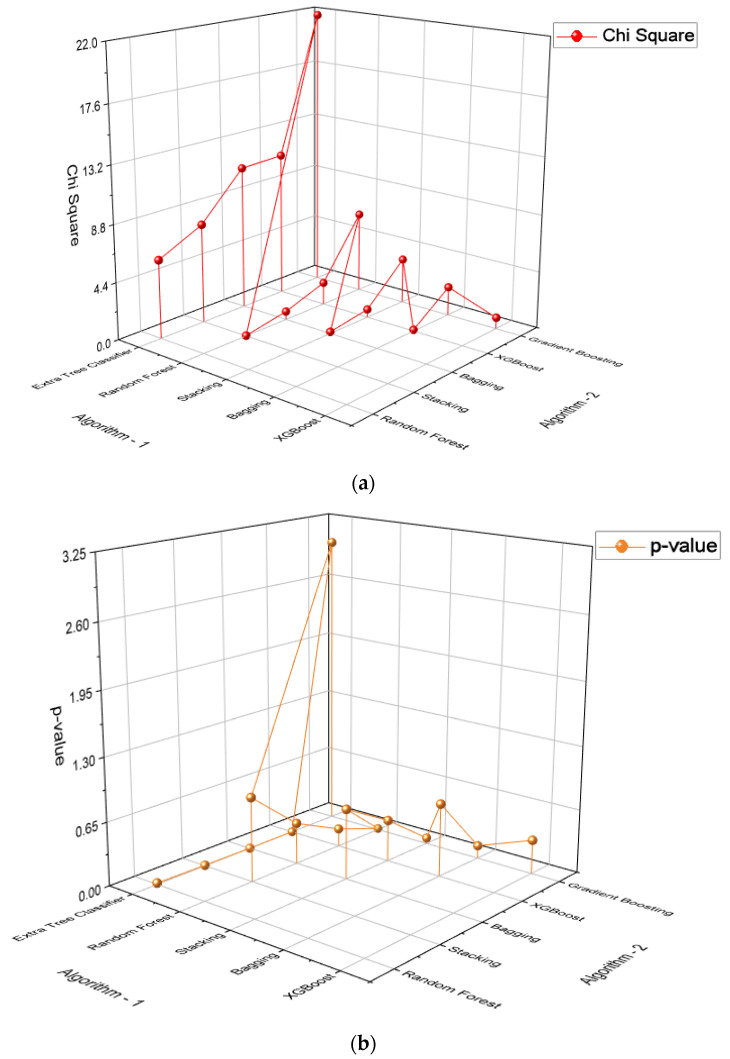
McNemar’s test between the models. (**a**) Chi-squared test. (**b**) *p* value test.

F-test for multiple classifier comparison was performed between the models. The p-value obtained for the test is 0.01856 which is lesser than significance level (α = 0.05). This denotes that we can reject the null hypothesis and conclude that there is a difference between the classification accuracies [39]. Since the null hypothesis was rejected, McNemar’s statistical test has been performed to find out which model pairs have different population proportions. McNemar’s test has a low false positive rate and is relatively fast to compute compared to other statistical tests [40]. If the p-value obtained for this test is less than significance level α = 0.05, we reject the null hypothesis that the two model perform equally. The results of the test have been tabulated in Table 6 and visualized in Figure 10. From the table, it can be concluded that the extra tree classifier has significant differences in performance when compared with most of the models.

#### 5.3.2. Visualization of Features

**Figure 11:**
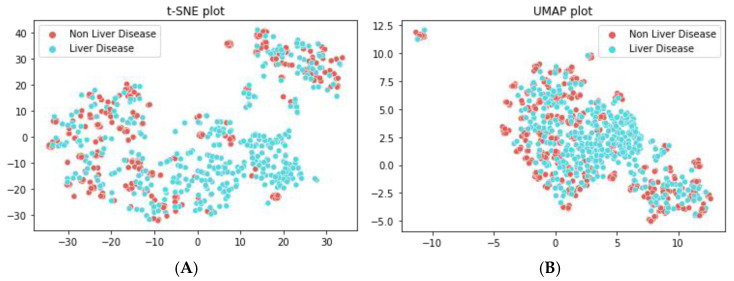
Feature visualization of extra tree classifier model. (**A**) t-SNE plot (**B**) UMAP plot.

Uniform manifold approximation and projection (UMAP) and t-distributed stochastic neighbor embedding (t-SNE) have been used to better understand the performance of the models on liver disease classification. t-SNE and UMAP are used to map high-dimensional features to two dimensions, enabling clear visualization of the data. In Figure 11A, the features with the final classification representation are depicted. Figure 11A shows the features well classified into liver and non-liver disease by the extra tree classifier model. The UMAP analysis was conducted to provide a more detailed representation of the features of the data. The distinction between the liver and non-liver class of proteins can be clearly observed in the UMAP plot in Figure 11B. Both the t-SNE and UMAP plots have effectively demonstrated the strong performance of the proposed model in accurately identifying liver disease.

### 5.4. Performance Comparison

**Figure 12:**
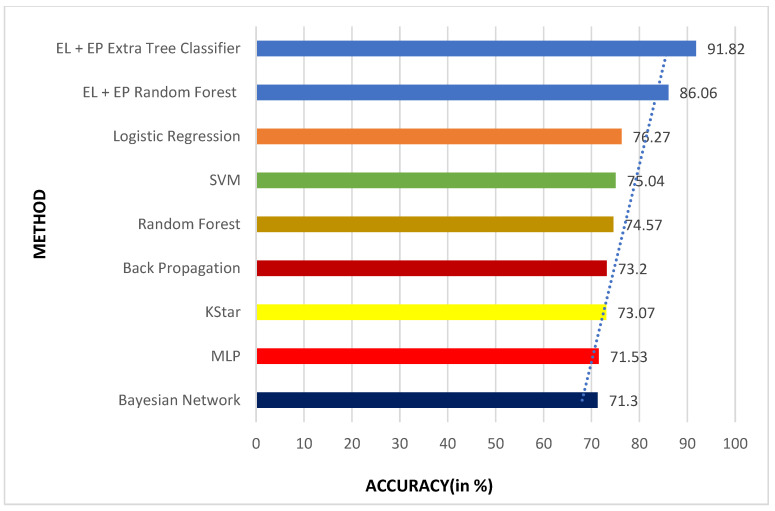
Comparison graph of proposed work with other existing works.

The performance of the ensemble algorithms used for liver disease classification is compared with existing works that have used the same dataset and evaluation methods. The results of the proposed work outperform many of the existing works. This is compared in Table 7 and Figure 12. The extra tree classifier shows the best results followed by the Random Forest model. The method proposed in this work uses enhanced preprocessing and ensemble machine learning and surpasses various other research works. Most of the other research works are based on simple machine learning models. Among them, Bendi et al. obtained an accuracy of 73.07% using the k star model, which is still low. The random forest accuracy for the proposed work is 86.06% and is much better than the results obtained by Sivakumar et al. for the same model. Overall, the extra tree classifier, which has not been used for liver disease classification before, surpasses all the other works with an accuracy of 91.82%.

## 6. Conclusions

Liver disease has been increasing annually in people across the globe. This is mainly due to lifestyle changes, and bad eating and drinking habits. Early diagnosis can help save people’s lives. To address this issue, several ensemble models have been used for liver disease diagnosis and their performance have been compared with other models. It was observed that the proposed model which uses enhanced preprocessing approach with extra tree classifier obtained the best testing accuracy of 91.82% followed by 86.06% for the random forest model. These proposed models outperformed many machine learning algorithms for liver disease classification present in the literature. This research was carried out on the ILPD dataset. For future work, different datasets can be integrated to carry out liver disease classification. This will help in increasing the training data and may improve the model accuracy further. Apart from that, better preprocessing methods and newer machine learning models such as C5.0, CBR (Case-based reasoning), and AODE (Aggregating One-Dependence Estimators) can also be trained on these datasets in the future.

## Figures and Tables

**Table 1:** Characteristics of patients.

Characteristics		Patients					
		All		With Liver Disease		Without Liver Disease	
		Number	%	Number	%	Number	%
Patients Enrolled		583	100	416	71.36	167	28.65
Age (in years)	Median	45		46		40	
	Range	4 to 90		7 to 90		4 to 85	
Gender	Male	441	75.64	324	77.88	117	70.06
	Female	142	24.36	92	22.12	50	29.94
Total Bilirubin (TB)	Median	1		1.4		0.8	
	Range	0.4 to 75		0.4 to 75		0.5 to 7.3	
Direct Bilirubin(DB)	Median	0.3		0.5		0.2	
	Range	0.1 to 19.7		0.1 to 19.7		0.1 to 3.6	
Alkaline Phosphotase(AP)	Median	208		229		186	
	Range	63 to 2110		63 to 2110		90 to 1590	
Alamine Aminotransferase (SGPT)	Median	35		41		27	
	Range	10 to 2000		12 to 2000		10 to 181	
Aspartate Aminotransferase (SGOT)	Median	42		52.5		29	
	Range	4 to 4929		11 to 4929		10 to 285	
Total Proteins (TP)	Median	6.6		6.55		6.6	
	Range	2.7 to 9.6		2.7 to 9.6		3.7 to 9.2	
Albumin	Median	3.10		3.00		3.4	
	Range	0.9 to 5.5		0.9 to 5.5		1.4 to 5.0	
Albumin and Globulin Ratio	Median	0.93		0.90		1	
	Range	0.3 to 2.8		0.3 to 2.8		0.37 to 1.9	

**Table 2:** Hyperparameter optimization for all the ensemble learning models.

Ensemble Models	Ranges of Hyperparameters	Optimal Value
Random Forest	n_estimators: [100, 150, 200, 500]	100
	criterion: [gini, entropy]	gini
	min_samples_split: [1.0, 2, 4, 5]	2
	min_samples_leaf: [1, 2, 4, 5]	1
	max_leaf_nodes: [4, 10, 20, 50, None]	None
Extra Tree Classifier	n_estimators: [100, 150, 200, 500]	100
	criterion: [gini, entropy]	entropy
	min_samples_split: [1.0, 2, 4,5]	2
	min_samples_leaf: [1, 2, 4, 5]	1
	max_leaf_nodes: [4, 10, 20, 50, None]	None
XGBoost	n_estimators: [100, 200, 500]	500
	learning_rate: [0.01, 0.05, 0.1]	0.05
	booster: [gbtree, gblinear]	gbtree
	gamma: [0, 0.5, 1]	0
	reg_alpha: [0, 0.5, 1]	0
	‘reg_lambda’: [0.5, 1, 5]	0.5
	‘base_score’: [0.2, 0.5, 1]	0.2
Gradient Boosting	‘n_estimators’: [100, 200, 500],	200
	‘learning_rate’: [0.1, 0.2, 0.5],	0.5
	‘criterion’: [‘friedman_mse’, ’mse’, ‘mae’],	friedman_mse
	‘min_samples_split’: [2, 4, 5],	2
	‘min_samples_leaf’: [1, 2, 4, 5]	1
Bagging	‘n_estimators’: [100, 200, 300]	200

**Table 3:** Proposed models evaluation metrics with respect to accuracy, precision, recall and specificity.

Algorithm	Accuracy (95% CI)	Precision (95% CI)	Recall (95% CI)	Specificity (95% CI)
Extra Tree Classifier	91.82 (87.88–95.19)	92.72 (87.50–97.17)	91.89 (86.54–96.43)	91.75 (85.44–95.48)
Random Forest	86.06 (81.25–90.38)	91.00 (85.00–96.04)	81.98 (74.54–88.50)	90.72 (84.27–95.79)
Stacking	85.10 (80.29–89.44)	80.76 (73.55–87.50)	94.59 (90.10–98.15)	74.22 (64.83–82.05)
Bagging	84.13 (78.85–88.47)	89.79 (83.33–95.56)	79.27 (71.31–86.33)	89.69 (82.95–95.40)
XGBoost	82.21 (76.92–87.50)	83.63 (76.72–90.27)	82.88 (75.73–90.09)	81.44 (73.33–88.79)
Gradient Boosting	78.85 (73.08–84.13)	83.83 (76.29–90.91)	74.77 (66.09–82.24)	83.50 (76.19–90.39)

**Table 4:** Proposed models evaluation metrics with respect to f1-score, roc_auc, 10-fold cross validation accuracy.

Algorithm	F1-Score	ROC_AUC	10-Fold Cross Validation Accuracy
Extra Tree Classifier	92.30	91.82	89.91
Random Forest	86.25	86.35	85.93
Stacking	87.13	84.41	93.15
Bagging	84.21	84.48	85.21
XGBoost	83.25	82.16	85.81
Gradient Boosting	79.04	79.13	80.41

**Table 5:** Anova F-test scores for all features with target feature.

S. No.	Features	Scores
1	DB	129.48
2	TB	121.47
3	SGOT	98.36
4	SGPT	92.54
5	AP	83.92
6	A/G	54.79
7	ALB	31.55
8	Age	13.34
9	Gender	03.69
10	TP	00.29

**Table 6:** McNemar’s test between the models.

Algorithm 1	Algorithm 2	Chi-Square	*p*-Value
Extra Tree Classifier	Random Forest	6.05	0.0139
Extra Tree Classifier	Stacking	7.6818	0.0055
Extra Tree Classifier	Bagging	11.1304	0.0008
Extra Tree Classifier	XGBoost	11.2813	0.0008
Extra Tree Classifier	Gradient Boosting	21.8064	3.0158
Random Forest	Stacking	0.0294	0.8638
Random Forest	Bagging	0.64	0.4237
Random Forest	XGBoost	1.75	0.1859
Random Forest	Gradient Boosting	6.3226	0.0119
Stacking	Bagging	0.1290	0.7194
Stacking	XGBoost	0.625	0.4292
Stacking	Gradient Boosting	3.5122	0.0609
Bagging	XGBoost	0.1026	0.7488
Bagging	Gradient Boosting	2.25	0.1336
XGBoost	Gradient Boosting	0.8780	0.3487

**Table 7:** Comparison of proposed work with other existing works.

S. No.	Source	Algorithm	Accuracy (in %)
1	Bendi et al. [7]	Bayesian Network	71.30
2	Bendi et al. [7]	MLP	71.53
3	Bendi et al. [7]	KStar	73.07
4	Sumedh et al. [12]	Back Propagation	73.2
5	Srivenkatesh et al. [40]	Random Forest	74.57
6	Geetha et al. [19]	SVM	75.04
7	Srivenkatesh et al. [40]	Logistic Regression	76.27
8	Ensemble Learning (EL) With Enhanced Preprocessing (EP)	Random Forest	86.06
9	Ensemble Learning (EL) With Enhanced Preprocessing (EP)	Extra Tree Classifier	91.82

## Data Availability

Not applicable.
